# Pharmacokinetic/pharmacodynamic optimization of temocillin treatment against CTX-M-15-producing *Klebsiella pneumoniae* isolates in a hollow-fiber infection model

**DOI:** 10.1128/aac.00946-25

**Published:** 2025-11-12

**Authors:** José Luis Rodríguez-Ochoa, Carlos Saúco-Carballo, Patricia Pérez-Palacios, Vicente Merino-Bohórquez, Ana Velázquez-Escudero, Lorena López-Cerero, Jesús Rodríguez-Baño, José Manuel Rodríguez-Martínez, Álvaro Pascual, Fernando Docobo-Perez

**Affiliations:** 1 Unidad Clínica de Enfermedades Infecciosas y Microbiología, Hospital Universitario Virgen Macarena 16582, Seville, Spain; 2 Instituto de Biomedicina de Sevilla IIIS, Hospital Universitario Virgen Macarena/CSIC/Universidad de Sevilla, Seville, Spain; 3 Centro de Investigación Biomédica en Red en Enfermedades Infecciosas (CIBERINFEC), Instituto de Salud Carlos III, Madrid, Spain; 4 Unidad de Gestión de Farmacia Hospitalaria, Hospital Universitario Virgen Macarena 16582, Seville, Spain; 5 Departamento de Farmacología, Facultad de Farmacia, Universidad de Sevilla 16778, Seville, Spain; 6 Departamento de Microbiología, Facultad de Medicina, Universidad de Sevilla 16778, Seville, Spain; 7 Departamento de Medicina, Facultad de Medicina, Universidad de Sevilla 16778, Seville, Spain; University of Fribourg, Fribourg, Switzerland

**Keywords:** temocillin, WGS, hollow-fiber

## Abstract

Temocillin, a β-lactam antibiotic, has been proposed as an alternative to carbapenems for the treatment of ESBL-producing Enterobacteriaceae. The present EUCAST breakpoints for temocillin are established based on exposure levels derived from a dosing regimen of 2 g every 8 h via intermittent infusion (II). However, the evidence supporting the efficacy of continuous infusion (CI) against ESBL-producing *Klebsiella pneumoniae* remains limited. The objective of the present study was to evaluate whether CI improves temocillin activity compared with II in a hollow-fiber infection model (HFIM) against CTX-M-15-producing *K. pneumoniae*. Four clinical isolates were characterized by whole-genome sequencing and tested for temocillin susceptibility. Mutant frequencies were estimated, and temocillin activity was assessed via time–kill assays and HFIM simulating human pharmacokinetics of temocillin 6 g/day via II and CI. Bacterial counts were performed to detect total and resistant subpopulations. Isolates showing regrowth were sequenced to identify resistance-associated mutations. The results showed that both regimens reduced bacterial burden within 8 h. However, three isolates regrew under II, while only one did under CI. CI achieved more sustained bacterial suppression and delayed or prevented the emergence of resistant subpopulations. Mutations in the *cpxA* gene were associated with increased temocillin MICs in regrown isolates. While both regimens demonstrated initial bactericidal activity, CI was more effective in sustaining bacterial suppression and limiting resistance emergence. These findings support the potential clinical benefit of CI for treating infections caused by CTX-M-15-producing *K. pneumoniae* and warrant further clinical validation.

## INTRODUCTION

Enterobacterales are a frequent source of serious infections in both the community and healthcare facilities. Resistance to widely used β-lactam antibiotics like penicillins and cephalosporins is largely due to extended-spectrum β-lactamases (ESBLs) (1). The treatment challenge is complicated by the fact that ESBL-producing bacteria often possess genes for resistance to other drug classes, including fluoroquinolones and aminoglycosides. In opposition to these isolates, studies, such as the MERINO trial, substantiate the benefits of carbapenems over other therapeutic options, including piperacillin/tazobactam, thus resulting in an augmentation in their utilization (2). In a scenario of elevated antibiotic pressure with carbapenems, it is anticipated that the number of resistant isolates will escalate, as has been previously observed in recent years (3–5). Consequently, the quest for alternative drugs that are efficacious against third-generation cephalosporin-resistant isolates is imperative. Temocillin is an old beta-lactam developed in the 1980s. Its use was limited due to its narrow therapeutic spectrum, as it is inactive against *Pseudomonas aeruginosa, Acinetobacter baumannii,* Gram-positive bacteria, and anaerobes. However, its resistance to hydrolysis by class A and C beta-lactamases makes it a potential option against ESBL- and AmpC-producing enterobacteria (6, 7).

EUCAST currently defines temocillin susceptibility breakpoints ([I] susceptible, increased exposure) for its use in infections originating from the urinary tract, against *E. coli, Klebsiella* spp. (except *K. aerogenes*), and *P. mirabilis* at a dose of 2 g every 8 h in intermittent infusion (II) (8). Since the pharmacodynamic parameter that best predicts the efficacy of beta-lactams is the time during which plasma concentrations are above the MIC of the isolate (*f*T > MIC), continuous infusion (CI) might optimize temocillin’s activity. This has been demonstrated for other beta-lactam antibiotics (9, 10), and also recently for temocillin against ESBL-producing *E. coli* (11). However, evidence of the advantages of CI against ESBL-producing *Klebsiella pneumoniae* remains scarce.

Therefore, the aim of the present study is to compare II versus CI in the treatment of ESBL-producing *K. pneumoniae* in a dynamic *in vitro* hollow-fiber infection model (HFIM).

## MATERIALS AND METHODS

### Bacterial isolate characterization

Four clinical isolates of ESBL-producing *K. pneumoniae* were selected for analysis, exhibiting different temocillin MIC values within the range of temocillin susceptibility (C6, C7, C9, and C10), from the collection of clinical isolates submitted to the Reference Laboratory for the Surveillance and Control of Nosocomial Infections and Prudent Use of Antimicrobials Program in Andalucía (PIRASOA program, Hospital Universitario Virgen Macarena Seville Spain). Molecular typing and characterization of the antimicrobial-resistant determinants were performed by whole-genome sequencing using MiSeq system (Illumina, San Diego, CA, USA). DNA sample library was prepared using Nextera XT DNA library preparation kit (Illumina). Raw reads were quality filtered and assembled *de novo* using CLC Genomic Workbench v10 (Qiagen). The annotation of the antimicrobial resistance determinants was carried out using ResFinder 4.1 (http://genepi.food.dtu.dk/resfinder) and CARD (12). Resistance determinant genes were assigned when 100% of homology and coverage was found.

### *In vitro* susceptibility studies

Temocillin MIC values were determined according to EUCAST guidelines using the broth microdilution method (ISO 20776-1). Temocillin (Negaban, Eumedica) concentrations ranged from 0 to 512 mg/L, and *E. coli* ATCC25922, as control strain, was used. The assays were conducted by three biological replicates. Susceptibility categories were assigned using EUCAST clinical breakpoints (8).

### Bacterial subpopulation analysis

The frequency of temocillin-resistant subpopulations was evaluated for the four selected clinical isolates. Briefly, an overnight culture of each strain (~10^9^ CFU/mL) was diluted 1:10^6^ to avoid the presence of mutants in the initial culture. Then, an initial inoculum of 10^3^ CFU/mL was incubated overnight on Mueller-Hinton broth (MHB). Next, 100 µL aliquots were properly diluted and plated on drug-free plates (to calculate the total bacterial concentration) and on Muller-Hinton agar (MHA) plates temocillin-containing. Mutants were isolated from MHA plates supplemented with temocillin at concentrations of 16 mg/L (approximately the minimum concentration, Cmin, observed in II) 32 mg/L (approximately the Cmin observed in CI) and 64 mg/L (corresponding to twofold dilutions of the current EUCAST susceptibility breakpoint). Plates were incubated for 24 h at 37°C. The assays were performed in triplicate. The frequency will be determined as the ratio between the number of colonies able to grow on antibiotic-containing medium and the total population.

### Temocillin activity in static assays

The temocillin activity was evaluated in time-kill assays. An initial inoculum of 1 × 10^6^ CFU/mL prepared and plated 96 deep-well plates (working volume of 2 mL) containing concentrations of temocillin ranging from 0.25 to 512 mg/L (in Log_2_ dilutions). A growth control (no temocillin) and a no growth control (no inoculum) were used. CFU/mL was determined at 0, 2, 4, 8, and 24 h by plating properly diluted samples onto free-drug MHA plates. The plates were incubated for 18 h at 37°C, and the lower limit detection was set at 2 log_10_ CFU/mL. The assays were performed in duplicate. A bactericidal activity was defined as a ≥3 log_10_ reduction in the initial inoculum (13).

### Temocillin activity in a dynamic infection model

In addition, temocillin activity was assessed in a HFIM mimicking the human temocillin pharmacokinetic profiles of two different dosing regimens, intravenous administration of 2 g/q8h in a 30 min infusion (II) and continuous infusion with 2 g loading dose followed by 6 g daily (CI). Temocillin stability of the CI was assessed with no degradation observed (data not shown) within the 24 h administration period (14).

The activity of temocillin against the four ESBL-producing clinical isolates of *K. pneumoniae* was assessed in a dynamic *in vitro* HFIM using polyethersulfone hollow-fiber cartridges (Aquamax HF03, Nikkiso, Belgium) along 3 days at 37°C. The unbound temocillin concentration (free fraction of 0.41) and time profiles were adjusted to mimic those observed in human plasma after II and CI (15). Based on *in silico* simulations in SIMULX (Monolix Suite, Lixoft), antimicrobial concentration-time profiles were performed in the HFIM by adjusting flow rates to replicate target concentrations and half-lives (t_1/2_ of 5 h). Two hours after each drug infusion, the rate of the pump controlling drug clearance was reduced to mimic the biphasic elimination of temocillin from plasma. Initial inoculum was set at 10^6^ CFU/mL, by inoculation of the extra-capillary space of each HFIM, with 50 mL of bacterial suspension. Bacterial concentrations were determined at 0, 2, 4, 6, 8, 24, 28, 48, 52, and 72 h. For total and resistant bacterial count, samples were plated on both drug-free and drug-containing (64 mg/L, fourfold susceptibility breakpoint) MHA plates. The lower limit detection was 2.0 log_10_ CFU/mL. Plates were incubated for 17 h at 37°C for subsequent counting of CFU/mL, and the assays were performed in duplicate.

### Temocillin-resistant mutant characterization

WGS were performed to detect the appearance of mutations of the isolates recovered from the HFIM. The original and post-HFIM isolates were processed, as previously described, and analyzed with Snippy v 4.6.0 via Galaxy Europe (https://github.com/tseemann/snippy) for the identification of genome variants related to temocillin resistance.

The wild-type *E. coli* BW25113 strain and the single-gene deletion mutant Δ*cpxA* were obtained from the KEIO collection to further characterize the role of *cpxA* inactivation in temocillin resistance (16). The temocillin MIC was then compared between the wild-type strain and the mutant to assess the effect of the mutation on temocillin susceptibility.

To explore the potential role of OXA-1 in temocillin resistance, a hyperproducing OXA-1 *E. coli* BEL21 strain was constructed by cloning a blaOXA-1 gene into a pET plasmid under the control of a pLAC promoter. The temocillin MIC was then compared between this plasmid-carrying strain and the plasmid-free isolate to assess whether the MIC increased.

### Drug assay for pharmacokinetics

The temocillin concentration was determined within the first dosing interval and at steady state. One milliliter was drawn from the central compartment on days 1–2 at selected time points (0, 2, 4, 6, 8, 24, 28, 48, 52, and 72 h). Samples were stored immediately at −80°C until analysis.

Temocillin concentrations were measured using a liquid chromatograph associated with a diode array ultraviolet spectrophotometer (HPLC-DAD, Agilent 1260 Infinity, Waldbronn, Germany) as described by Miranda et al. with modifications (17). The validated working concentration range was linear from 1 to 100 µg/mL (R^2^ > 0.9998), the accuracy was within 96%–101%, and the coefficients of variation in intraday and interday precision were less than 10% and within the precision range of 95%–105%. The low limit of quantification (LLOQ) was 1 µg/mL.

## RESULTS

### *In vitro* susceptibility studies and bacterial subpopulation analysis

**Fig 1:**
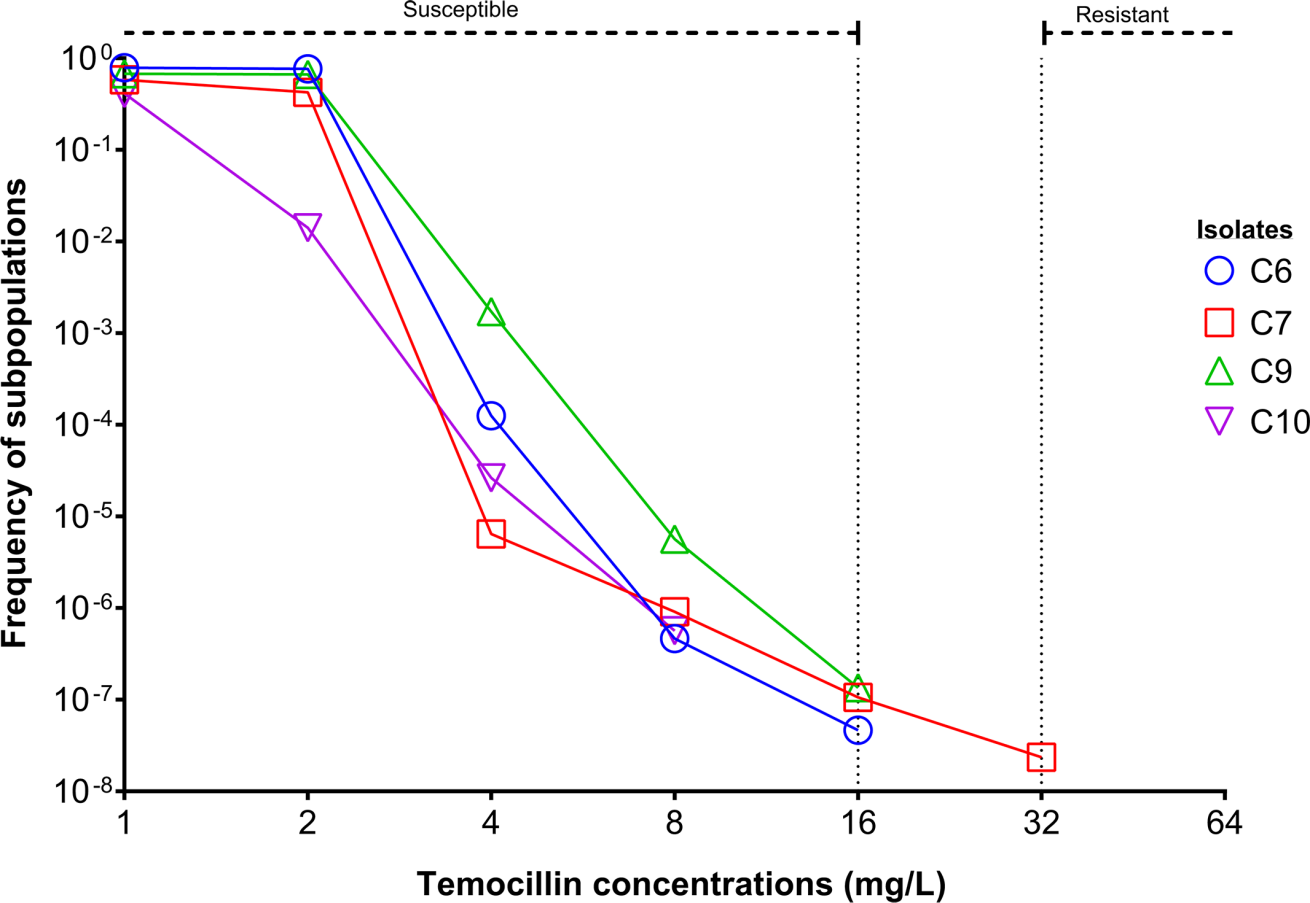
Population analysis profiling (PAP) of C6, C7, C9, and C10 plated on the indicated temocillin concentrations. The proportion of total colonies was calculated compared to growth on drug-free plates. EUCAST breakpoint ranges are highlighted at the top of the figure.

Temocillin MIC values and *bla* gene contents are shown in Table 1. All isolates were susceptible with increased exposure (I). Regarding the possibility of detecting resistant subpopulations (Fig. 1), no mutants were detected at 16 mg/L (Cmin observed in II) for C10 (frequency ≤2.8·10^−9^) strains . However, colonies of C6 and C9 were obtained at frequencies of 4.7·10^−8^ and 1.4·10^−7^, respectively. At 32 mg/L (Cmin observed in CI), mutants were detected only for the C7 strain (frequency of 2.4·10^−8^). No mutants were recovered from any of the clinical isolates tested at temocillin concentrations of 64 mg/L.

**TABLE 1:** Characteristics of CTX-M-15-producing *K. pneumoniae* isolates and the percentage of time that the free drug concentration remained above multiples of the MIC (%*f*T_>MIC_)

Isolate	β-lactamase genes	Temocillin MIC (mg/L)	PKPD index %ƒT > MIC					
			1× MIC		4× MIC		8× MIC	
			II	CI	II	CI	II	CI
C6	*bla*_CTX-M-15_ *, bla*_TEM-1_	4	100	100	48.8	100	17.5	91
C7	*bla*_CTX-M-15_ *, bla*_TEM-1_ *, bla*_OXA-1_	4	100	100	48.8	100	17.5	91
C9	*bla*_CTX-M-15_ *, bla*_TEM-1_	16	100	100	0	0	0	0
C10	*bla*_CTX-M-15_ *, bla*_OXA-1_	8	100	100	17.5	100	0	0

### Temocillin time-kill assays

**Fig 2:**
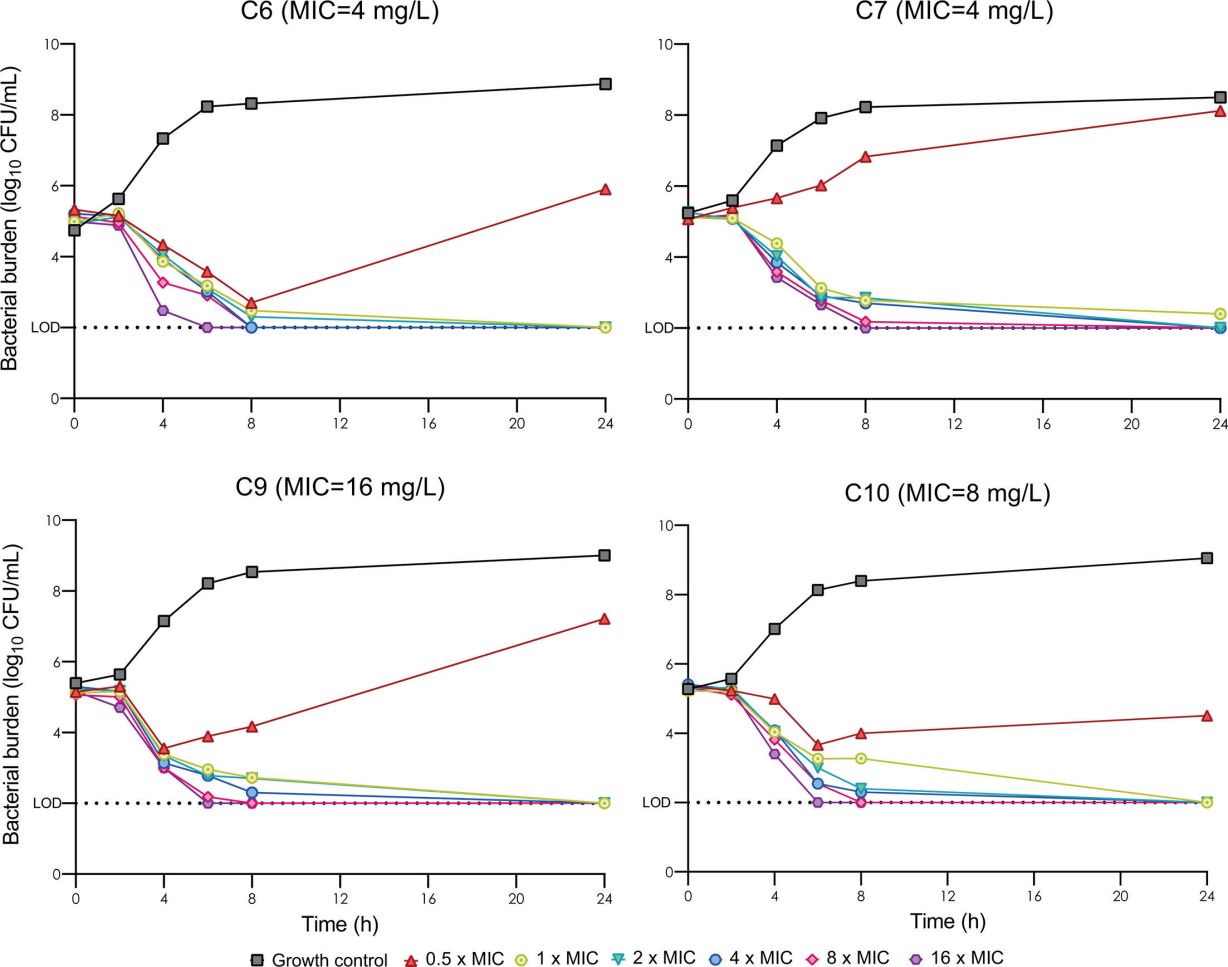
Bacterial population over 24 h at temocillin concentrations ranging from 0.5 to 16 times the MIC for each isolate. The yellow line represents bacterial population in MHB at temocillin concentration equal to isolate MIC.

The results of the time-kill curves are shown in Fig. 2. At temocillin concentrations of 0.5 times the MIC for each isolate, C6, C9, and C10 strains showed a reduction in burden, with no total eradication or regrowth after 24 h. No isolate was able to grow at concentrations below its MIC value. For concentrations equal to or above each MIC value, a reduction of 3 log_10_ was observed for all isolates within the first 8 h, with bacterial counts below the limit of detection after 24 h, except for C7 at 1× MIC.

### Hollow-fiber infection model

#### Temocillin pharmacokinetics and pharmacodynamics

**Fig 3:**
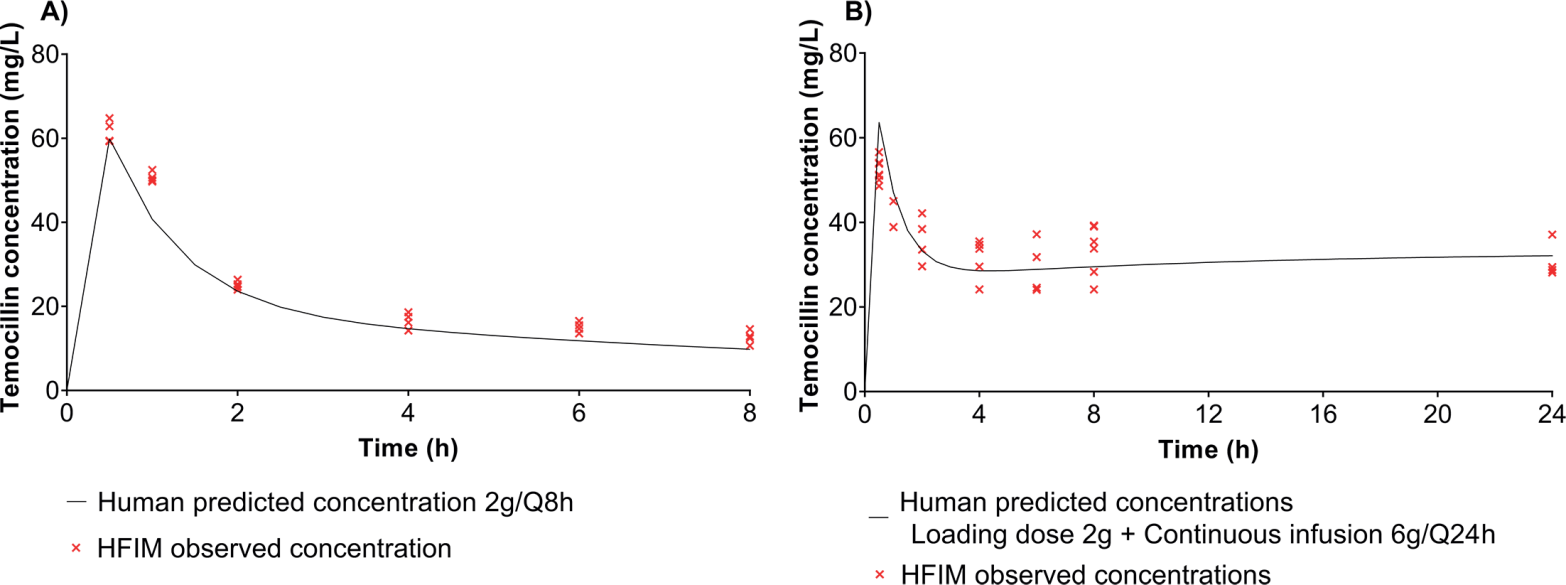
Observed versus human-like temocillin concentrations in the hollow-fiber infection model experiments after (**A**) 30 min intermittent infusion of 2 g q8h and (**B**) loading dose of 2 g followed by continuous infusion of 6 g q24h.

The observed versus predicted concentrations of temocillin concentrations in the HFIM after temocillin II and CI dosages are shown in Fig. 3. Temocillin-simulated PK data were within 10% range of the target concentrations. Both administrations of temocillin resulted in a PD exposure of 100% *f*T  > MIC of the dosing interval for all isolates (Table 1). With regard to the II exposure, it was observed that almost 50% of the dose interval exceeded 4× MIC for C6 and C7, and 17.8% for C10. No exposure was noted for C9. However, 100% of the dose interval was covered by CI for C6, C7, and C10. Finally, only CI was able to show a %*f*T > 8× MIC above 90% of the dose interval for C6 and C7.

#### Effect of temocillin on total bacterial population

**Fig 4:**
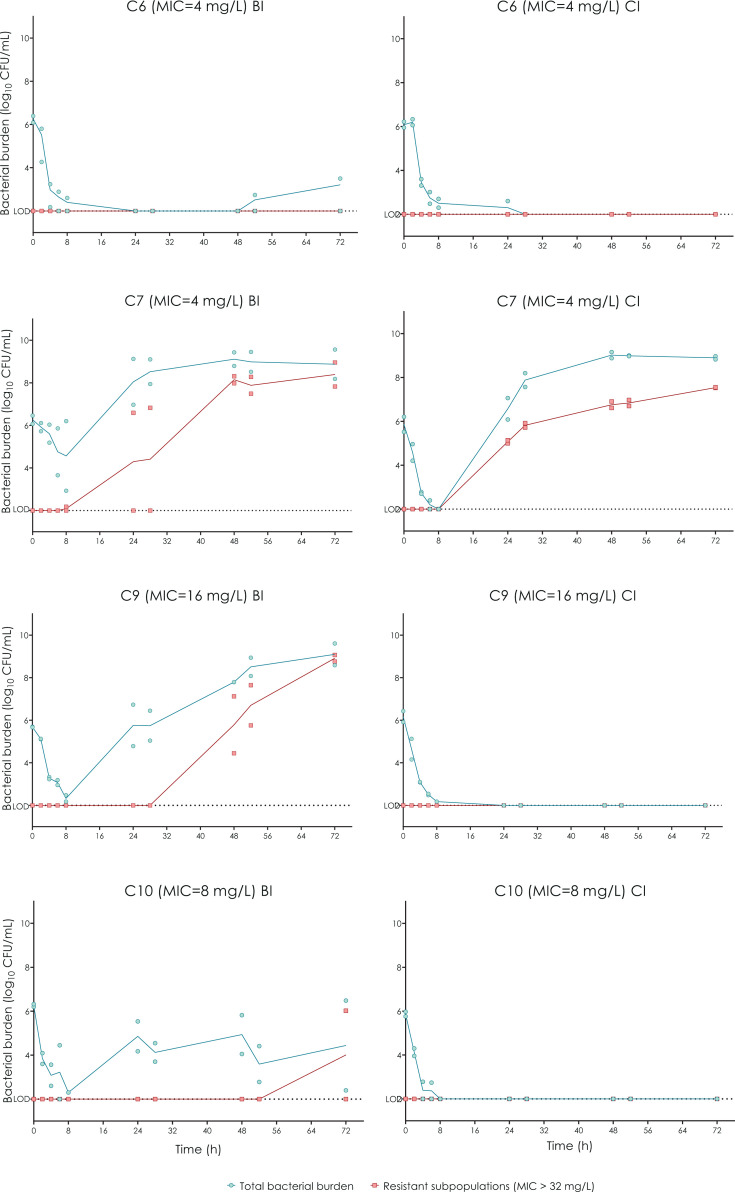
HFIM of CTX-M-15-producing *K. pneumoniae* mimicking a temocillin dosage in II (30 min intermittent infusion of 2 g q8h, left panel) and CI (loading dose of 2 g followed by continuous infusion of 6 g q24h, right panel). Circle and square symbols denote total and temocillin-resistant bacterial populations, respectively. The limit of detection (LOD) was set at 2 log_10_ CFU/mL.

Bacterial concentrations over the course of the HFIM experiment under both administration regimens are presented in Fig. 4. Intermittent infusion achieved a reduction of approximately four logs_10_ in the total bacterial burden for isolates C6, C9, and C10 within the first 8 h of treatment. A reduction of the initial inoculum was observed for isolate C7 too, though in this case, it was approximately 2 logs_10_. Following this initial reduction, isolates C7, C9, and C10 demonstrated regrowth after 24 h of treatment. C7 and C9 reach maximum population after 48 h and C10 regrowth is approximately 2 logs_10_ in the final point. A slight regrowth of less than 2 logs_10_ was observed at 48 h for isolate C6 (in a single replicate).

On the other hand, all isolates showed an initial inoculum reduction of approximately 4 log 10 with the CI dosing, with complete eradication of the inoculum being achieved at 24 h for C9 and C10 and at 28 h for C6. In the case of C7, after the initial reduction, regrowth was observed at 24 h, reaching the maximum population at 48 h.

#### Effect of temocillin on emergence of resistance

Resistant subpopulations were observed in three of the four isolates after II: at 24 h of treatment in C7, at 48 h in C9, and at 72 h in C10. These resistant subpopulations reached the total bacterial burden for all three isolates at the last time point of the experiment. In the case of CI administration, the appearance of these resistant subpopulations was only observed for isolate C7, emerging 24 h after the initial treatment. The MIC values for each resistant subpopulation are shown in Table 2. Among the isolates recovered from the HFIM treated with temocillin II, isolates C7 and C9 showed mutations in the *cpxA* gene: (i) isolate C7 of assay 1, disruptive in-frame deletion 45_50delCACGCT [Thr16_Leu17del] and of assay 2, missense variant 322T > G [Phe108Val]); and (ii) isolate C9 of assay 1, missense variant 125A > T [Glu42Val] and *acrD* missense variant 521A > T [Asp174Val] and of assay 2 conservative in-frame insertion 53_58dupCGCTGG [Ala18_Leu19dup]). No mutations were detected for C10.

**TABLE 2:** Initial and final temocillin MIC values after HFIM assay exposed to temocillin II or CI

Isolate	Original MIC (mg/L)	Bacterial subpopulation after HFIM assay Temocillin MIC (mg/L)	
		Intermittent infusion of 2 g q8h (II)	Loading dose of 2 g + continuous infusion of 6 g q24h (CI)
C6	4	4^*a*^	NR^*b*^
C7	4	256	≥512
C9	16	≥512	NR
C10	8	128	NR

^*a*^ Strain obtained in a single replicate assay.

^*b*^ NR, no regrowth.

Regarding the isolates recovered from the HFIM treated with temocillin CI, C7 showed a mutation in the *cpxA* gene (missense variant 322T > G [Phe108Val]) and in the *acrD* gene (missense variant 322T > G [Phe108Val]) in one assay and a mutation in the *cpxA* gene (missense variant 2988C > A [Ser996Arg]) in the replicate.

### Impact of *cpxA* or *bla*_OXA-1_ hyperexpression in temocillin resistance

In order to understand the mechanisms underlying the augmented temocillin resistance observed after the HFIM assays, an isogenic model was employed. Temocillin MIC of the wild-type *E. coli* BW25113 strain was determined to be 4 mg/L, in contrast to the 16 mg/L observed for the Δ*cpx* single-gene deletion mutant. Conversely, no alterations in temocillin MIC were detected in the OXA-1-hyperproducing *E. coli* BEL21 strain.

## DISCUSSION

Although a growing number of studies support the efficacy of temocillin against ESBL-producing Enterobacteriaceae (11, 18, 19), little is known about the way to optimize temocillin activity against ESBL-producing *K. pneumoniae*. The results of this study demonstrate two strengths of CI over II: first, greater and more sustained reductions in bacterial inoculum are achieved over time, and, second, CI is more effective in preventing the emergence of resistant subpopulations.

It is also noteworthy that temocillin displays high bactericidal activity in the presence of hydrolysis resistance by ESBL. This could be observed in the time-kill assay, where bactericidal activity was observed at temocillin concentrations equal to MIC for all isolates. These findings align with those obtained in the hollow fiber model, demonstrating levels approaching the limit of detection within 8 h of initiating treatment for all isolates, except for C7 in II. This could be partly explained by the difference in inoculum between the two assays. Both assays used an inoculum of 10^6^ CFU/mL. However, the time-kill assays used a volume of 2 mL, while the HFIM model used 50 mL of bacterial culture. This implies that there is a 25-fold difference in inoculum in HFIM compared with the time-kill assays, which could lead to a higher probability of the presence of subpopulations with decreased susceptibility in the HFIM model and therefore lower activity.

However, none of the isolates were completely eradicated at the end point of the study when II was administrated. In contrast, CI achieved complete eradication of the bacterial inoculum in three of the four isolates. In a separate HFIM study, Van Os et al. demonstrated that continuous infusions and 6 g daily doses of temocillin exhibited superior efficacy against ESBL-producing *E. coli* in comparison to 4 g daily doses and intermittent infusions (11). While these results are limited to the HFIM setting, large clinical trials (e.g., BLING-III) and meta-analyses have also suggested a potential advantage of prolonged or continuous infusion of β-lactam antibiotics in critically ill patients, although the observed effect sizes are generally modest (20). For instance, Abdul-Aziz et al., in a recent meta-analysis including 18 randomized clinical trials with 9,108 critically ill adults with sepsis or septic shock, reported that prolonged infusion of β-lactams was associated with a reduced risk of intensive care unit mortality (risk ratio, 0.84; 95% credible interval, 0.70–0.97; high certainty) and an increased likelihood of clinical cure (risk ratio, 1.16; 95% credible interval, 1.07–1.31; moderate certainty) (9, 11).

Regrowth observed in the HFIM was mainly due to the emergence of resistant mutants. For isolate C6, regrowth occurred after 52 h without detectable resistant subpopulations. In the cases of isolates C9 and C10, the appearance of resistant subpopulations was delayed by a minimum of 24 h, indicating the possibility that these subpopulations may have emerged during an extended assay. Although CI allows better PD parameters to be achieved, its role in reducing the emergence of resistant mutants has not been validated. Dhaese et al. did not find a benefit of continuous infusion over intermittent infusion for piperacillin/tazobactam or meropenem in terms of suppression of emergence of antimicrobial resistance in ICU patients treated with these drugs (21). In our study, the mutant prevention concentration (MPC) was not determined, but considering that the minimum plasma concentration in CI is more than twice that in II (37 and 16 mg/mL, respectively), it could explain the lower emergence of resistance observed. It is possible that this MPC is exceeded in CI and not in II.

Isolates C7 and C10 expressed, in addition to CTX-M-15, an OXA-1 oxacillinase. While OXA-like enzymes are generally associated with temocillin resistance because they are not hydrolyzed by class D β-lactamases (22–24), this is not the case for OXA-1, whose expression does not confer temocillin resistance (25). Both isolates were able to regrow (C9 only under II and C7 under both conditions). To further evaluate the role of OXA-1 in temocillin resistance, a hyperproducing OXA-1 pET plasmid was expressed in an *E. coli* BEL21 background, which resulted in no significant changes in temocillin MIC.

The low mutation frequency values for all isolates are consistent with those previously reported (26). Notably, with the exception of isolate C10, the isolates in which pre-existing mutants were detected, and at the concentrations where they were detected, correspond to those that were capable of regrowth in the HFIM: C7 and C9 under the II regimen and in the temocillin-resistant mutant frequency study at 16 mg/L (≈II C_min_), and C7 under the CI regimen and in temocillin-resistant mutant frequency study at 32 mg/L (≈CI C_min_). However, the initial bacterial inoculum in the HFIM experiment was lower than the observed mutant frequency for each isolate, so regrowth is unlikely to be due to the presence of pre-existing temocillin-resistant subpopulations.

In this study, isolates C7 and C9, which were capable of regrowth, exhibited missense mutations in *cpxA* after the HFIM. The *CpxA/CpxR* system is a two-component regulatory system that activates in response to cell envelope stress. Mutations in *cpxA* lead to nonspecific activation of this pathway, one consequence of which is the overexpression of the *acrD* efflux pump (27). Guerin et al. demonstrated that *acrD* overexpression increased temocillin MIC values by 8- to 16-fold (28). To the best of our knowledge, mutations in the *cpxA* gene have not previously been proposed as a mechanism of temocillin resistance in *K. pneumoniae*. Nevertheless, such mutations have been observed in *Serratia marcescens*, resulting in an increase in temocillin MIC from 4 to 1,024 mg/L (29). Similar systems that impacted *acrD* expression have been described. In the study by Guerin et al.*, acrD* overexpression was driven by a single mutation in *baeS*. The *baeS/baeR* system, another two-component regulatory system, is similarly activated by cell envelope stress. Previous research by this group found that mutations in *baeS* could result in an eightfold increase in MIC values (30). Furthermore, in a prior HFIM experiment examining the efficacy of II against *KPC-2*-producing *K. pneumoniae*, mutations in *baeS* were also observed in isolates capable of regrowth (31). The twofold increase in temocillin MIC observed in the *cpxA* knockout strain compared with the wild-type supports the hypothesis that the elevated MICs in these isolates are, at least in part, attributable to the loss-of-function mutations identified in this gene.

This study has certain limitations. The hollow-fiber design has the intrinsic limitation of the absence of the immune system and the use of an artificial broth. In the presence of an effective immune response, bacterial proliferation may be inhibited. Indeed, following a substantial reduction in bacterial load resulting from antimicrobial action, the immune system may be capable of eradicating residual bacteria, thereby preventing the regrowth of susceptible or resistant strains. This would explain the lack of correlation between the eradication rate in this *in vitro* study and that observed *in vivo*. In this sense, Van Os et al. recently conducted HFIMs, including ESBL-producing *E. coli* isolates and the same temocillin dosing of the present study, showing this same regrowth effect by the emergence of resistant mutants (11). In this study, all isolates were able to regrow at the endpoint in the II posology with a 0% eradication rate. However, a retrospective cohort study demonstrated that temocillin achieved an overall microbiological cure rate of 84% in infections caused by ESBL/dAmpC-producing Enterobacteriaceae when administered in a 2 g/q12 h posology, lower than that used in this study (18). Additionally, a comparative study between temocillin and carbapenems used for the treatment of urinary tract infections caused by ESBL-producing Enterobacteriaceae reported a clinical cure rate of 94% for temocillin, which was not significantly different from the 99% cure rate observed with carbapenems (19). Finally, although no mutations were detected in isolate C10, we cannot rule out the possibility of an unstable resistance mechanism that may have been reversed during the processing of isolates without antimicrobial pressure. In this regard, further insights into the mechanisms of temocillin resistance might have been obtained by analyzing resistant subpopulations from PAP or time-kill experiments.

In conclusion, the achieved exposure after 2 g q8h of temocillin in II showed an extensive initial bacterial killing *in vitro* against CTX-M-15-producing *K. pneumoniae* strains. However, CI achieved more extensive bacterial suppression than II, thereby preventing the emergence of highly resistant temocillin subpopulation. Taken together, our findings, along with previous HFIM studies and animal data, support the concept that CI may offer advantages over II in terms of sustained suppression of bacterial growth and limiting resistance. However, we believe that the current evidence, while compelling, is insufficient to recommend a universal shift to CI in clinical guidelines. Dedicated randomized trials with temocillin are unlikely due to feasibility constraints, but pragmatic observational studies and real-world stewardship programs could provide valuable evidence, particularly in severe infections caused by ESBL-producing *K. pneumoniae*. In our view, CI can be considered a reasonable option in selected clinical scenarios where high bacterial burden or risk of resistance emergence is anticipated, while further clinical validation is required before guidelines can endorse CI as standard of care.
